# Omics Approaches for Engineering Wheat Production under Abiotic Stresses

**DOI:** 10.3390/ijms19082390

**Published:** 2018-08-14

**Authors:** Tariq Shah, Jinsong Xu, Xiling Zou, Yong Cheng, Mubasher Nasir, Xuekun Zhang

**Affiliations:** 1 Key Lab of Biology and Genetic Improvement of Oil Crops, Oil Crops Research Institute, Chinese Academy of Agricultural Sciences (CAAS), Wuhan 430062, China; zouxiling@gmail.com (X.Z.); chengyong58@139.com (Y.C.); 2 College of Natural Resources and Environment, Northwest Agriculture and Forestry University, Yangling 712100, China; mubasher@mwsuaf.edu.cn

**Keywords:** abiotic stresses, GWAS, ionomics, omics, phenomics, QTL

## Abstract

Abiotic stresses greatly influenced wheat productivity executed by environmental factors such as drought, salt, water submergence and heavy metals. The effective management at the molecular level is mandatory for a thorough understanding of plant response to abiotic stress. Understanding the molecular mechanism of stress tolerance is complex and requires information at the omic level. In the areas of genomics, transcriptomics and proteomics enormous progress has been made in the omics field. The rising field of ionomics is also being utilized for examining abiotic stress resilience in wheat. Omic approaches produce a huge amount of data and sufficient developments in computational tools have been accomplished for efficient analysis. However, the integration of omic-scale information to address complex genetics and physiological questions is still a challenge. Though, the incorporation of omic-scale data to address complex genetic qualities and physiological inquiries is as yet a challenge. In this review, we have reported advances in omic tools in the perspective of conventional and present day approaches being utilized to dismember abiotic stress tolerance in wheat. Attention was given to methodologies, for example, quantitative trait loci (QTL), genome-wide association studies (GWAS) and genomic selection (GS). Comparative genomics and candidate genes methodologies are additionally talked about considering the identification of potential genomic loci, genes and biochemical pathways engaged with stress resilience in wheat. This review additionally gives an extensive list of accessible online omic assets for wheat and its effective use. We have additionally addressed the significance of genomics in the integrated approach and perceived high-throughput multi-dimensional phenotyping as a significant restricting component for the enhancement of abiotic stress resistance in wheat.

## 1. Introduction

Wheat is the 3rd most cultivated cereal crop throughout the globe (Acevedo et al. 2002) covering 22% of the cultivated land. It belongs to the family of the Gramineae (Poacea). It mainly grows in the temperate zones with cool weather (12–25 °C temperature) and having 250–1750 mm annual precipitation [1]. In case of winter wheat, it is widely planted at the end of autumn season as it needs the cold treatment for flower initiation, which is called vernalization process [2]. A recessive allele of Vrn genes on 5A, 5B and 5D genomes is responsible to control this growth habit of winter wheat, which requires 40 °C temperature prior to tillering and elongation stage [2,3]. Both genotype and photoperiod are major factors to control the flowering pattern in wheat, which lack photoperiod insensitivity genes (Ppd) need exposure to long growing days [4].

Abiotic stress is one of the key factors limiting the crop production. Environmental stresses like drought stress, salt stress, heavy metal stress as well as water submergence stress influenced the crop yield in a different manner. To overcome such problems, local cultivars should be modified by making molecular changes in specific genes [5]. Although the multi-selection field trial method has been widely used for direct selection of tolerant varieties for any harsh environment, this selection method does not provide significant results for the abiotic stress related traits that are highly influenced by environmental conditions and low heritability [6]. In addition, this direct selection approach is quite laborious as well as time consuming. The genetic variation in different yielding crops could lead to the development of tolerant cultivars but there is a knowledge gap which needs strong effort to find specific molecular markers [7]. The development of molecular markers will provide the new and latest sequenced genomes and organelles in crops [8].

Recently, the development of molecular markers to characterize the complete genome sequence plays a crucial role in marker-assisted breeding [9]. The availability of high density markers helps to identify different alleles involved in agronomic traits and also helps in haplotype analysis [10]. In addition, marker-assisted breeding has been accomplished for the simple traits, which are controlled by a single or few loci [11,12] however, such breeding also suffers due to unsought genetic strains [11]. The phenotypic expression of the newly identified genes is controlled by the genetic makeup of the repeated parents, which is mainly due to epistatic interaction [13] and this epistatic interaction in mostly unpredictable in case of multiple complex traits, unless some proper evidence is available about the molecular processes involved during the developments of new traits. Current improvement in the genomics can easily predict the factors involved in their genetic variation, traits developments, distribution as well as interaction with the host environment [14]. Genetic Engineering is an advanced approach mainly used for the genetic enhancement of the plants. Interestingly, genetically modified (GM) plants have been proven to be successful for herbicide and insect resistance and widely used throughout the world [15]. A combination of multi-disciplinary knowledge is required for the development of an ideal plant, which could provide better yield even in adverse climatic conditions. This review has been written to explain the recent achievements in various “omics” approaches and to elaborate the future outcomes for the development of abiotic stress tolerant varieties.

Advances in technology have additionally given a high-throughput, effective and quick array-based genotyping stages. The single nucleotide polymorphism SNP array advancement requires starting data about SNPs, luckily, data around a large number of SNPs is now accessible in the general population space (Table 1). The Illumina Infinium array (SoySNP50K iSelect BeadChip) for ~50,000 SNPs has been effectively created and utilized for the genotyping of a few soybean plant presentation (PI) lines [16]. Advances in technology beyond this make it conceivable to resequence several lines in a cost effective way and has begun a new era of genotyping by re-sequencing [17]. Presently, the challenge for plant scientists is how to successfully utilize these assets for marker-assisted applications.

## 2. Omics Approaches in the Technological Era

**Figure 1:**
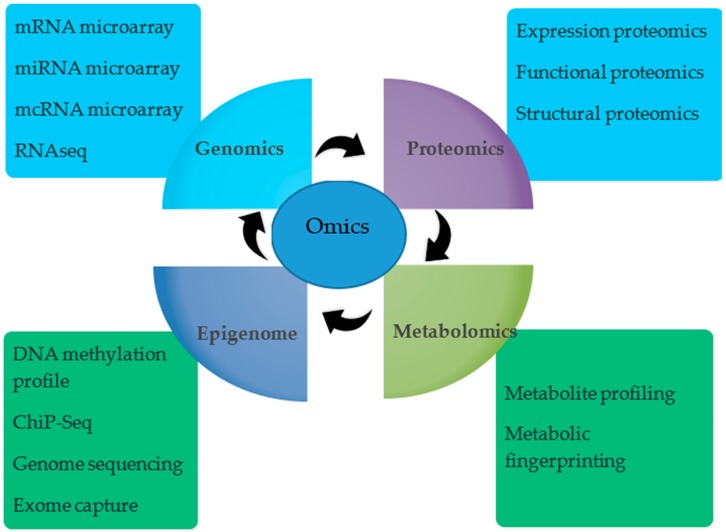
Key branches of omics and their major components being used at molecular and genetic level in different integrated approaches in wheat.

When genetics, nutritional or environmental conditions are changed a diverse mix of technologies fulfil the understanding of the changes that occur. Omics are useful in the understanding of species and thus providing insight into modification of the plant metabolism which results through contact with environment. The era of genomics had been started with the development of automated sequencing methods and led to first whole genome sequencing of *Arabidopsis thaliana*. The genome sequencing has been stretched out to major crop plants such as rice [18,19], soybean [20], maize [21]. The emergence of high throughput “Omics” approaches has begun a successful period of plant molecular techniques for adjusting to changes in the environment. Recent development in ‘omics’ after post genomic era such as next generation sequencing, genome scale molecular analysis, modeling of different physiological and molecular understanding and correlation of these observations with physiology of the plant provides an accomplished move to adaptability and productivity under stress. Latest advent of next generation sequencing methods made possible sequencing the plant species quite useful [22]. Allohexaploid bread wheat (2n = 6x = 42, AABBDD genomes) is one of the most complicated genomes in which homologous chromosomes having similar genes complicates the reconstructing process of biological networks. A draft of the wheat genome is completed which shows more than 124,000 gene loci which covers all the sub-genomes (A, B and D) and proves useful in identifying genes which control biological process. Further, modern utilization of transcriptomics (RNA-seq) and proteomics (targeted vs. non-targeted proteins) will help in defining their functions at the gene and protein level respectively. As all genes are not always turned on at the same time, therefore the metabolism becomes quite dynamic in phenotype which cannot be derived from the genotype. Thus, the successful integration of the transcriptomics (gene), proteomics (proteins), metabolomics (metabolite), ionomics (analysis of elemental compositions), epigenomics (inheritance), interactomics (protein-protein or protein-DNA interactions) will facilitate the breeder to select the potential candidates and best traits to generate and improve the crop productivity under abiotic stress (Figure 1).

## 3. Genomics Progresses for Abiotic Stress Tolerance in Wheat

Genomics emphasis on the genome physical structure, aiming to recognize, detect and order genomic structures along chromosomes. Here we discuss some of genomic progresses to understand abiotic stress tolerance in wheat.

### 3.1. Molecular Marker Resources

The emergence of genomic technology has opened a new window in genetic enhancement of complicated traits such as salt and drought tolerance. The amalgam of genomic approaches along with marker assisted selection (MAS) can be helpful in the identification of specific genes at a much faster rate in the breeding population as compared to classical breeding [23,24,25,26]. Developments in MAS for wheat has lagged behind other crops due to limited genomic data but recent progress in DNA sequencing and genotyping techniques have developed genome datasets which are very useful in designing sequence based simple sequence repeats (SSRs) and SNP markers [27,28,29]. SNPs are frequently used for genome mapping and germplasm characterization as compared to other molecular markers. As SNPs are high-throughput, rapid, cost-effective, co-dominant, sequence tagged and highly abundant, they are appropriate for division of complex traits using highly multiplexed marker microarrays such as the Affymetrix GeneChip [29,30]. For instance, Axiom Wheat Breeders’ genotyping array, a robust system for screening large wheat population, is developed recently. It is a cost effective and efficient genotyping method having 35,143 pre-validated SNPs which covered all wheat chromosomes and have the ability to genotype 384 samples at once. In durum wheat, it has been applied recently in the development at linkage maps of high density and also in identifying the genomic areas of complex traits such as drought tolerance [31].

### 3.2. Quantitative Trait Loci (QTL) Mapping for Abiotic Stress

For agronomically important traits, linkage maps are necessary for mapping the QTLs as they are constructed from genotype data from multiplexed marker assays [32,33,34,35]. High-density linkage maps also offer a genomic resource for positional cloning of significant genes. They can also be applied in comparative genomics to assess the chromosomal organization and evolution as they are constructed from sequence tagged markers. In the linkage map, markers are helpful in identifying regions having QTLs of selected traits and several QTLs have mapped previously for salt tolerance [17,35,36] and drought tolerance [37,38,39] in wheat cultivars (Table 1).

Advances in genomics and phenomics provide us more precise and broad characterization of the QTLs that control a targeted trait known as QTLome. The vast knowledge on QTLome put a responsibility on breeders to utilize this knowledge in an effective way. Enhanced QTL meta-analysis, valuation of QTL effects and upgraded crop modeling will allow an actual utilization of the QTLome [40].

### 3.3. Genome Wide Association Studies

**Figure 2:**
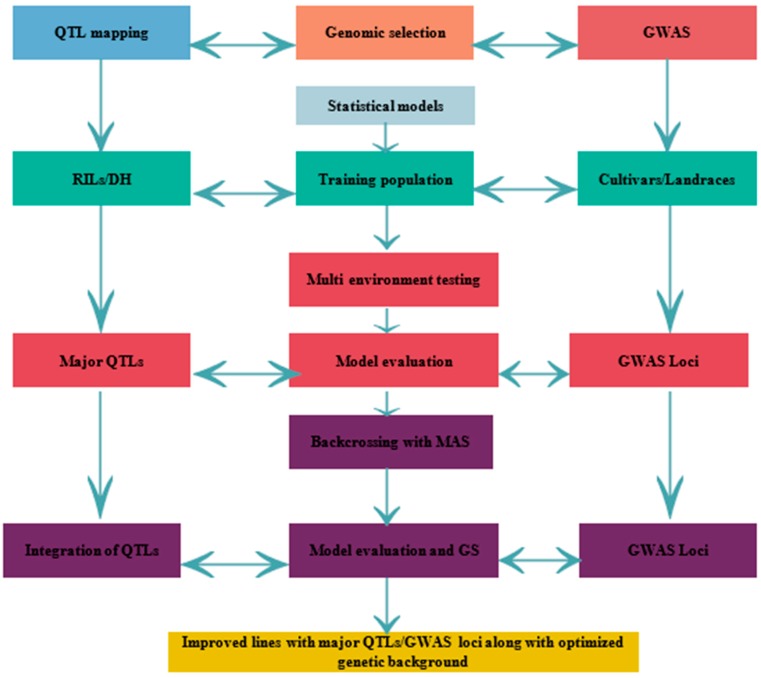
A combined approach of QTL mapping/Genome-wide association study (GWAS) and Genomic selection (GS). Schematic diagram of genomics-assisted breeding. Genomics technologies help enhancing marker trait association for marker-assisted selection (MAS) and genomic selection (GS). Both MAS and GS speedup selection cycles, increases precision and improves genetic gain per year. Selection and recombination will be duplicated multiple times before the yield trials to increase the favorable allele frequency. Incorporation of genomics to the recurrent selection strategies substantiates the effectiveness of the breeding program.

Another approach to overcome these limitations is association mapping that uses the historical meiosis in the diversity panel and provides more precision [51]. It is quite feasible and cost effective to establish an association mapping in compared to recombinant inbred line (RIL) development. In association mapping, experimental structures and statistical evaluation are constantly fluctuating to diminish the results of confounding factors, decrease false positives and also control minor allele effects (Figure 2). Genetic interaction and population designs confound marker-trait relations results in the disequilibrium without true linkages [52]. To decrease false positives and minor allele QTL effects, several diagnostic and precise statistical analyses have been developed. Studies have been carried out at the correct genomic locations of developmental genes such as reduced height (Rht), vernalization (Vrn) and photoperiod responsiveness (Ppd) and used as a standard measure in order to incorporate phenotypic diversity and markers present in the study [3]. As a genetic reference, these genes possess the stress adaptive capability by changes heading date, plant height, maturity and other physiological processes [53].

### 3.4. Genomic Selection

The emergence of model based association and easy availability of molecular markers, a correlated concept known as genomic selection has emerged to assess genotypes breeding value [54]. This technique is utilized to minimize the shortcomings of map based genetic analysis that identify very few QTL to explicate the divergence in targeted traits [54]. The concrete significance of evaluating the QTL effect and linkage disequilibrium based on the genetic relatedness and the divergence of the population under study. Populations that show the more allelic variation of targeted traits display more precise evaluation of QTL effect compared to populations which are more closely related. Linkage disequilibrium is often overvalued in closely related inter-mating individuals and diminish in further meiotic events [55]. Genomic breeding value can be predicted by genomic selection by transforming marker assisted selection with the help of markers. A model that is established and evaluated using genotypic and phenotypic data of study population will be utilized to assess phenotypic variation of sample population based on their genetic composition only. This will enhance genetic gain in comparison to both QTL and phenotype-based selection [56].

Statistical methods are used to develop genomic selection models that explain the properties of various markers and traits [57]. The distribution of marker effects and random sampling of germplasm from selected population is being considered by the multiple regression models [58]. Genomic best linear unbiased prediction evaluates genetic relatedness among individuals on the basis of molecular marker composition similarity and also assess their phenotypic performance. Such a model is similar to estimation of breeding value (BV) from heritability and phenotypic performance of related genotypes in a pedigree [54]. Statistical analyses show that forward and mixed type regression models have ability to remove markers on the basis of their relative significance effect. Ridge regression has an additional feature of incorporating penalty parameter in the design of the markers in excess of the statistically accepted number (>number of genotypes) [59].

### 3.5. Transcriptome Profiling for Abiotic Stress Tolerance

**Figure 3:**
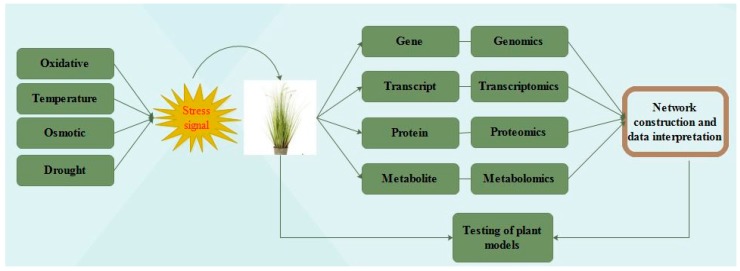
General outline of ‘omics’ approach for network construction, data interpretation and model testing. Schematic diagram of successful methods for the genome wide transcript expression profiling and is being widely used to generate transcriptional profiles.

In wheat, expressed sequence tag databases showed that homologous genes can show expression in one but remain silent in one or both of the remaining genomes in the analysis of gene expression [60]. For the cereals, various microarray and macroarray platforms have been developed. There are various significant arrays like 10,000 cDNA array reported by Leader [61] and Affymatrix arrays have been developed for wheat and barley recently [62]. In abiotic stress conditions in a variety of plants, the microarray is one of the successful methods for the genome wide transcript expression profiling and is being widely used to generate transcriptional profiles (Figure 3). Studies have been carried out in barely in response to salt stress [63,64] and the model cereal *Brachypodium* [65] but there is limited research on wheat due to its polyploidy genome [66]. The high-throughput analysis of gene expression is being enabled by deep genome sequencing technology (RNA-Seq) which proves to be a successful technique to identify precise changes in the genome. Next-generation sequencing technology is in the emerging phase in plant studies but it is predicted to replace the microarray technique due to its accurate results. Due to the lack of fully sequenced and complex genome (hexaploid) in wheat present many difficulties to “OMICS” studies. Meng et al. [67] assessed alkalinity stress by digital expression tag profiling method in wheat. Poersch-Bortolon et al. [68] assessed the extreme drought stress in roots and leaves by examining their expression profiles which prove to be helpful in marker development and selecting the significant important genes. These expression profiles are also proving useful in wheat drought stress by examining their metabolic processes. Ma et al. [69] conducted an RNA-seq analysis to analyze the effect of drought on the wheat genome during reproductive stages. Salt stress responsive gene networks and functional annotation were identified by differential root transcriptome analysis, which prove to be helpful in understanding the process and genes role in salt tolerance as analyzed by Goyal et al. [70].

## 4. Proteomics in Wheat

It is imperative to study proteome alterations at various stress conditions as proteins play a role in plant stress response. The initial plant response to stress conditions is the cellular processes of stress sensing and signaling processes. In order to understand the stress coping mechanisms in plants, effective characterization and isolation of stress responsive proteins is required. Understanding post-translational modification of proteins is necessary in plant stress conditions. Study of proteomics provides vast information on the fine-tuning of cellular pathways that once took part in stress mitigation. The significant data related to changes in response to abiotic stress and their major role in differential stress response are elaborated in Table 2.

## 5. Metabolomics Advances for Abiotic Stress

Metabolites provide the energy which is necessary for metabolism and growth of protoplasm as they are the basic elements of various structural and enzymatic molecules. Metabolites not only serve as a link between phenotype and genetic information but also play a role in determining the physiological condition of organism. Metabolomics along with other disciplines played a significant role in understanding the interrelated biological processes linked to phenotypes. In functional genomics, metabolic profiling is an emergent tool [92]. The co-existence of gene transcript and metabolites provides a foundation for generating data-driven theoretical models of the biological phenomenon [93,94,95]. In the study of mutants and transgenic lines, metabolomics not only help in understanding metabolism systems but also give information about candidate genes [96,97,98]. The metabolic study also explains how particular genes affect the metabolic pathway and its interaction with other pathways, which is not possible with other techniques such as microarray [99,100].

The advances in metabolomics, the availability of whole genome sequence, genome-wide genetic variants and cost-effective genotyping assays has opened exciting doors to blend metabolomics with crop breeding programs [101,102]. Mass spectrometry (MS) and nuclear magnetic resonance (NMR) spectrometry proved to be successful technologies in wheat. Metabolomics cover not only familiar metabolites but also unfamiliar metabolites as confirmed by wide scale metabolite assessments but managing the ample data is still a challenge [103,104]. Metabolic annotations can be progressed through metabolomics approaches when coupled with advance Bioinformatics techniques, for instance, in case of model plant *Arabidopsis* [105,106]. Genomic data can also be improved through the sequencing of DNA, RNA and MS quantification of proteins and metabolites which helped in the improving of targeted traits [107]. A number of studies have been conducted on agriculturally useful crops to evaluate the effect of salinity and drought on metabolic activity of the crop in studying stress responses [108,109]. The enhanced tolerance of particular genes may be due to different phenotypic responses which explains the specific metabolic changes. Improvement of breeding material and selection of superior traits can be facilitated through metabolomics approaches [110]. In the bread wheat genome, the polyploid nature is quite distinctive as it has the ability to tolerate considerable chromosomal aberrations and allowing phenotypic assessment as a result of multiple gene loss. Aneuploid genetic lines of wheat proved to be an effective experimental design for the assessment of genetic networks, as they are helpful in identifying those genes which control phenotypes [111,112]. Michaletti et al. [113] concluded that the study of metabolomics and proteomics reveal drought stress responses of leaf tissue from spring wheat and provide a better framework for understanding the mechanisms that control plant cell responses to drought stress while providing knowledge of molecules that can be used for crop improvement programs.

## 6. Ionomics for Wheat

Ionomics is a high-throughput elemental profiling method to study the molecular systematic basis essential mineral nutrient and trace element composition of living systems. It also represents the inorganic component of cellular and organismal systems [114]. Its wide role in forward and reverse genetics, screening of mutants, uncovering the mechanisms of ion uptake, compartmentalization, transport and exclusion are useful in understanding the processes of abiotic stresses in plants [115]. Ionomics along with relevant genomic data can identify the cellular changes during abiotic stresses as well as the basic processes involved. It can thus help in understanding the gene networks controlling the ion accumulation at different growth stages under abiotic stress. It is proving to be helpful in understanding the gene networks which control ion accumulation of varied growth steps under the conditions of abiotic stress [116]. The literature available on ionomics approaches is limited, however, the highly effective ion profiling will open new doors in understanding the signaling mechanisms for abiotic stress tolerance.

## 7. Phenomics Prospective in Wheat

Phenomics is the study of high-throughput phenotypic analysis. It has become possible to predict genotypes that are susceptible to abiotic stress through phenotyping approaches [117]. For high throughput plant phenotyping, the automated Greenhouse system is a successful procedure. This system permits the non-destructive screening of plants over a particular period with the help of image acquisition techniques. The different images of each plant are recorded and then evaluated using advanced image analysis algorithms to predict plants with certain phenotypes [118]. The plants with tolerant phenotypes proved to be good sources of genomic resources and also became targets for different molecular approaches including high-throughput sequencing to identify the alleles of interest. However, phenomics also has some disadvantages as it did not provide accurate correlation among the values obtained in the pot culture versus field experiment.

## 8. Role of Online Databases for Effective Integration of Omics Platforms

The emerging progress in omic approaches has established a huge amount of data which prove to be helpful in a number of research activities. Computational resources have helped to store, catalog and analyze data and make it easily available through user friendly “databases”.

A number of databases have been developed for wheat (Table 3). The most reliable database is (http://www.gramene.org) which gives wide information for omics data from a number of other sources. These databases provide useful information regarding wheat translational genomics and molecular breeding research. It comprises genes, proteins, microRNAs, sRNAs, metabolites, molecular markers and phenomic information of wheat plant introductions (PI). Integration of multi-omics data sets information is also presented in these databases. For example, genes in the QTL region can be retrieved very easily along with the functional annotations, associated protein information in respect of structure and functional features, syntenic information with other model plants, sequence variation among different cultivars, gene expression data including tissue specific variations and many other types of information for wheat.

## 9. Conclusions and Future Perspectives

Diverse omics tools have been utilized to understand how wheat plants react to abiotic stress conditions. We understand that the studies to integrate various omics approaches are restricted in wheat because of the expanded cost and potential challenging integrated omic scale investigation. Ongoing improvements in computational assets, statistical tools and instrumentation have brought down the cost of omics in the numerous folds, however integrated analysis needs novel devices and technical wizards. The complete idea of multiomic studies provides a totally new road and future research projects should plan to adjust appropriately. In wheat, genomics and transcriptomics have developed as expected but the other major omic branches like proteomics, metabolomics and phenomics are as yet lingering behind. These omic branches are similarly vital to get clear photo of the organic framework. Eminently, phenomic studies should be widely utilized along with alternate omics approaches. Desired phenotype is the ultimate aim of crop sciences; therefore, it needs to be understood intensely. Diverse omic tools and integrated methodologies examined in the present review will give looks of current situations and future viewpoints for the successful management of abiotic stress resilience in wheat.

A major focus in the future will be the combining and comparative analysis of transcriptome information between specialists. Specifically, there are huge assets of wheat microarray information now accessible, which give opportunities for gene co-expression network analysis. The merging of information got from a standardized platform, for example, the Affymetrix Wheat GeneChip, will be much easier than cross-platform merging. For instance, the authors of this review have partaken in four studies of the wheat transcriptional response to rust pathogens utilizing the Affymetrix Wheat GeneChip platform. The information from these four studies will be integrated to perform a gene expression network analysis that can possibly recognize key gene expression signatures engaged in rust resistance. This kind of analysis has been effective in associating abiotic stress phenotypes in *A. thaliana* with fundamental normal and unique gene expression pathways and marker genes [121]. The linking of gene expression information to genetic data is also of extraordinary significance to comprehend the interchange among genome, transcriptome and phenotype. Synchronous studying of the genome and transcriptome on a single platform through, for instance, the ELPs and SFPs discussed earlier, is a significant strategy for revealing genetic components that impact the transcriptome. In general, wheat transcriptomics is advancing quickly and later on we will see an entire systematical approach that coordinates the transcriptome with genome, proteome and metabolome. Furthermore, gene expression databases will expand and become more standardized, which will quicken the revelation and characterization of all genes in the wheat genome.

## Figures and Tables

**Table 1:** Major and stable quantitative trait loci (QTL) with percentages of explained variance (PVE) ranging from 19% to 59% for agronomic and physiological traits.

S.No.	QTL	Linked Markers	Position	Env. ^a^	PVE (R2) ^b^	References
Agronomic traits						
**A. Grain Yield**						
1	qGYWD.3B.2	Xgpw7774	97.6	7/4	19.6	[41]
2	4A	Xwmc420	90.4	Mean/2	20	[42]
3	4A-a	Xgwm397	6	7/5	23.9	[43]
4	Qyld.csdh.7AL	Xgwm322	155.9	21/11	20.0 *	[44]
**B. 1000-Grain weight**						
1	3B	Xbarc101	86.1	Mean/2	45.2	[45]
2	QTgw-7D-b	XC29-P13	12.5	11/10	21.9	[46]
**C. Days to Heading**						
1	QDh-7D.b	XC29-P13	12.5	11/11	22.7	[47]
2	QHd.idw-2A.2	Xwmc177	46.1	13/16	32.2	[46]
**D. Days to Maturity**						
1	QDm-7D.b	X7D-acc/cat-10	2.7	11/10	22.7	[48]
**Physiological Traits**						
**A. Stem Reserve Mobilization**						
1	QSrm.ipk-2D	Xgwm249a	142	2/2	42.2	[48]
2	QSrm.ipk-5D	Xfbb238b	19	2/2	37.5	[48]
3	QSrm.ipk-7D	Xfbb189b	338	2/2	21	[48]
**B. Water Soluble Carbohydrate**						
1	QWsc-c.aww-3A	Xwmc0388A	64.9	2/2	19	[49]
**C. SPAD/Chlorophyll Content**						
1	Qchl.ksu-3B	Xbarc68	67.2	3/2	59.1	[50]

^a^ Number of environments in which QTL was detected/number of total environments; ^b^ highest PVE (R2) values under drought/water stress, * with >20% higher yield per ear.

**Table 2:** A list of wheat proteomics studies focused on response to abiotic stresses and others.

Stress/Conditions	Treatment Time and Dose	Cultivar	Organ/Organelle	Proteomic Technologies	Stress Induced Modulation of Metabolic Pathways	Differentially Expressed Protein Classification		References
						Functions	Localizations	
Flooding	7 d	Bobwhite line SH 9826	Seminal root	2-DE, nano LC-MS/MS	Antioxidant defense	StrRes	-	[71]
Flooding	2 d	Shiroganekomugi	Root	2-DE, nano LC-MS/MS	Carbohydrate (Glycolysis)	EnMet, ProtMet, SigTran, Tranp	Cell wall	[72]
Drought	100 d	Opata, Nesser	Root	iTRAQ	Energy metabolism, Replication, Repair	EnStr, Oxired, Trans	Mem, Cyto, Cell wall, Mito, Nucl, Plast, Vacu	[73]
Drought	7 d	Ofanto	Leaf	2-DE, MALDI-TOF	Carbohydrate (Glycolysis, gluconeogenesis)	PTR, StrRes, TCA, ROSsca, AAB, GG	-	[74]
Drought	7 d	Katya, Sadovo, Zlatitza, Miziya	Leaf	SDS-PAGE, 2-DE	Energy (photosynthesis)	EnMet, EnvDevS	Chlo	[75]
Drought	9 d	Keumkang	Leaf	2-DE, MALDI-TOF/TOF	Energy (photosynthesis)	Photo	Chlo	[76]
Drought	10, 15, 20 and 25 d	Janz, Kauz	Seed	2-DE, MALDI-TOF	Carbohydrate metabolism	ROSsca, CarMet, SigTran	-	[77]
Drought	14, 24 d	Kukri, Excalibur	Leaf	iTRAQ	Energy (photosynthesis)	Photo, GG, ProtF, Tranp, EnStr	-	[78]
Drought	20% PEG	Hanxuan 10 and Ningchun 47	Leaf	nano LC-MS/MS	Antioxidant defense	DRM, SigTran, StrRes, ROSsca	-	[79]
Heat and Drought	10 d	Vinjett	Kernel	2-DE, MALDI-TOF	Carbohydrate (Glycolysis)	CarboMet, STP	-	[80]
High Temperature	37 °C d, 28 °C N/10 d, 20 d	Butte 86	Endosperm	2-DE, QSTAR PULSAR-TOF	Carbohydrate metabolism	CarboMet, NitMet, ProtMet, StrRes, STP, SigTran, Tranp, Trans	-	[81]
Salt	150 mM NaCl/1 d, 2 d, 3 d	Keumkang	Leaf	2-DE, LTQ-FTICR-MS	Energy (photosynthesis)	Photo, StrRes	Chlo	[82]
Salt	1.0, 1.5, 2.0 and 2.5% NaCl in HS/2 d	Zhenhmai 9023	Leaf	2D-DIGE/Q-TOF-MS	Carbohydrate metabolism	CarMet, ProtF, Tranp, ROS, ATP	-	[83]
Salt	200 mM	Wyalkatchem, Janz	Shoot	2-DE, LC-MS/MS	-	-	Mito	[84]
Aluminum	250 µM/2 d, 3 d	Atlas-66, Fredrick	Root	SDS-PGE, Immunoblot	Signaling pathway	Oxi	-	[85]
Aluminum	100, 150 µM/5 d	Keumkang	Root	2-DE, LTQ-FTICR-MS	Energy (Glycolysis)	Gly, Tranp, SigTran, StrRes, EnMet	-	[86]
Copper	100 µM/3 d	Yumai 34	Root, Leaf	2-DE, HPLC-Chip/ESI-Q-TOF/MS/MS	Energy (photosynthesis), antioxidant defense	StrRes, SigTran, ProtMet, CarMet, Photo, EnMet	-	[87]
Protein Profiling	20 d	Keumkang	Leaf	SDS-PAGE, LTQ-FTICR	Energy (photosynthesis)	COB, DevPro, DRM, ProtF, ProtMet, StrRes, Tranp, Trans	Chlo	[88]
Protein Profiling	Mature seed	Wild type (AA, BB, DD genome)	Seed	SDS-PAGE, nano LC-MS/MS	Carbohydrate metabolism	StrRes, EnMet, ProtS, CGD, COD, ProtF, SigTran, STP, Tranp	-	[89]
Cadmium	10, 100 and 200 µM	Yangmai 15	Leaf	IPG, MALDI-TOF	Energy (photosynthesis)	Oxi, ProtMet, Photo	-	[90]
Cadmium	0.5 mM/L	Yangmai 13	Leaf	IPG, MALDI-TOF	Antioxidant defense	ROSsca	-	[91]

AAB—Amino acid biosynthesis; ATP—ATP synthase; CarMet—Carbohydrate metabolism; CGD—Cell growth and division; COB—Cell organization; COD—Cellular organization and development; DevPro—Developmental process; DRM—DNA and RNA metabolism; EnMet—Energy metabolism; EnStr—Environmental stress; EnvDevs—Environmental and developmental signals; GG—Glycolysis and gluconeogenesis; Gly—Glycolysis; NitMet—Nitrogen metabolism; Oxi—Oxidative stress; OxiRed—Oxidation-reduction process; Photo—Photosynthesis; ProtF—Protein folding; ProtMet—Protein metabolism; ProtS—Protein synthesis; PTR—Post-transcriptional regulations; ROSsca—ROS scavenging; SigTran—Signal transduction; STP—Storage proteins; StrRes—Stress response; TCA—Calvin cycle; Tranp—Transport, Trans—Translation; Chlo—Chloroplast; Mem—Membrane; Cyto—Cytoplasm; Nucl—Nucleus; Mito—Mitochondria; Plast—Plastid; Vacu—Vacuole; HS—Hoagland solution; d: days.

**Table 3:** Online transcriptomics resources in wheat.

Resources	Description/URL
Genome sequence	Coordinated effort underway by the IWGSC (http://www.wheatgenome.org) Recognized as a priority by the research community (http://www.csrees.usda.gov/nea/plants/pdfs/wheat_conference_summary.pdf)
ESTs ^1^	1,050,791 entries
Oligonucleotide microarray	1,050,791 entries
cDNA microarray	Multiple including ~9 K array
Tiling microarray	Not currently available
Serial Analysis of Gene Expression (SAGE)	Applied for studying, developing wheat caryopsis
Massively Parallel Signature Sequencing (MPSS)	Not reported
Sequencing-by-synthesis	Roche 454 cDNA sequencing [119]
Deletion and aneuploid genetic stocks	Roche 454 cDNA sequencing [119]
Transformation	Biolistic- and Agrobacterium-mediated DNA delivery systems
Gene knockdown	RNA interference Viral-induced gene silencing
Databases/tools	Graingenes (http://wheat.pw.usda.gov) Gramene (http://www.gramene.org) TIGR Genome Database (http://www.tigr.org/tdb/e2k1/tae1) Wheat Genome Project (http://wheat.pw.usda.gov/NSF/htmlversion.html) Wheat Genome Project (http://wheat.pw.usda.gov/NSF/htmlversion.html) wEST (http://wheat.pw.usda.gov/wEST) CerealsDB (http://www.cerealsdb.uk.net) HarvEST Wheat (http://harvest.ucr.edu) PLEXdb (http://plexdb.org) Gene Expression Omnibus (http://www.ncbi.nlm.nih.gov/geo) ArrayExpress (http://www.ebi.ac.uk/microarray-as/ae) GrainSAGE (http://www.scu.edu.au/research/cpcg/igfp/index.php) Wheat SNP Project (http://wheat.pw.usda.gov/SNP/new/index.shtml) [120]

Esterase, ^1^ ESTs listed in the National Center for Biotechnology Information (NCBI) EST database (GenBank dbEST) (8 August 2008; http:// www.ncbi.nlm.nih.gov/dbEST/dbEST_summary.html).

## References

[1] Leonard W.H., Martin J.H. (1963). Cereal Crop.

[2] Santra D., Santra M., Allan R., Campbell K., Kidwell K. (2009). Genetic and molecular characterization of vernalization genes Vrn-A1, Vrn-B1 and Vrn-D1 in spring wheat germplasm from the Pacific Northwest region of the U.S.A. Plant Breed..

[3] Gomez D., Vanzetti L., Helguera M., Lombardo L., Fraschina J., Miralles D.J. (2014). Effect of Vrn-1, Ppd-1 genes and earliness per se on heading time in Argentinean bread wheat cultivars. Field Crop. Res..

[4] Goncharov N.P. (2003). Genetics of growth habit (spring vs. winter) in common wheat: Confirmation of the existence of dominant gene Vrn4. Theor. Appl. Genet..

[5] Grainger C.M., Rajcan I. (2014). Characterization of the genetic changes in a multi-generational pedigree of an elite Canadian soybean cultivar. Theor. Appl. Genet..

[6] Manavalan L.P., Guttikonda S.K., Phan Tran L.S., Nguyen H.T. (2009). Physiological and molecular approaches to improve drought resistance in soybean. Plant Cell Physiol..

[7] Xu X., Liu X., Ge S., Jensen J.D., Hu F., Li X., Dong Y., Gutenkunst R.N., Fang L., Huang L. (2012). Resequencing 50 accessions of cultivated and wild rice yields markers for identifying agronomically important genes. Nat. Biotechnol..

[8] Tomar R.S.S., Deshmukh R.K., Naik K., Tomar S.M.S., Vinod. (2014). Development of chloroplast−specific microsatellite markers for molecular characterization of alloplasmic lines and phylogenetic analysis in wheat. Plant Breed..

[9] Song Q., Jia G., Zhu Y., Grant D., Nelson R.T., Hwang E.Y., Hythen D.L., Cregan P.B. (2010). Abundance of SSR motifs and development of candidate polymorphic SSR markers (BARCSOYSSR_1. 0) in soybean. Crop Sci..

[10] Tardivel A., Sonah H., Belzile F., O’Donoughue L.S. (2014). Rapid identification of alleles at the soybean maturity gene E3 using genotyping by sequencing and a haplotype-based approach. Plant Genome.

[11] Shi A., Chen P., Li D., Zheng C., Zhang B., Hou A. (2009). Pyramiding multiple genes for resistance to soybean mosaic virus in soybean using molecular markers. Mol. Breed..

[12] Jun T.H., Mian M.R., Kang S.T., Michel A.P. (2012). Genetic mapping of the powdery mildew resistance gene in soybean PI 567301B. Theor. Appl. Genet..

[13] Palloix A., Ayme V., Moury B. (2009). Durability of plant major resistance genes to pathogens depends on the genetic background, experimental evidence and consequences for breeding strategies. New Phytol..

[14] Morrell P.L., Buckler E.S., Ross-Ibarra J. (2011). Crop genomics: Advances and applications. Nat. Rev. Genet..

[15] Carpenter J.E. (2010). Peer-reviewed surveys indicate positive impact of commercialized GM crops. Nat. Biotechnol..

[16] Song Q., Hyten D.L., Jia G., Quigley C.V., Fickus E.W., Nelson R.L., Cregen B.P. (2013). Development and evaluation of SoySNP50K, a high-density genotyping array for soybean. PLoS ONE.

[17] Xu Y., Li S., Li L., Zhang X., Xu H., An D. (2013). Mapping QTLs for salt tolerance with additive, epistatic and QTL× treatment interaction effects at seedling stage in wheat. Plant Breed..

[18] Goff S.A., Ricke D., Lan T.H., Presting G., Wang R., Dunn M., Glazebrook J., Sessions A., Oeller P., Varma H. (2002). A draft sequence of the rice genome (*Oryza sativa* L. ssp. *japonica*). Science.

[19] Yu J., Hu S., Wang J., Wong G.K.S., Li S., Liu B., Deng Y., Dai L., Zhou Y., Zhang X. (2002). A draft sequence of the rice genome (*Oryza sativa* L. ssp. *indica*). Science.

[20] Schmutz J., Cannon S.B., Schlueter J., Ma J., Mitros T., Nelson W., Hyten D.L., Song Q., Thelen J.J., Cheng J. (2010). Genome sequence of the palaeopolyploid soybean. Nature.

[21] Schnable P.S., Ware D., Fulton R.S., Stein J.C., Wei F., Pasternak S., Liang C., Zhang J., Fulton L., Graves T.A. (2009). The B73 maize genome: Complexity, diversity and dynamics. Science.

[22] Weckwerth W. (2011). Green systems biology—From single genomes, proteomes and metabolomes to ecosystems research and biotechnology. J. Prot..

[23] Castillo A., Budak H., Varshney R.K., Dorado G., Graner A., Hernandez P. (2008). Transferability and polymorphism of barley EST-SSR markers used for phylogenetic analysis in *Hordeum chilense*. BMC Plant Biol..

[24] Vogel J.P., Tuna M., Budak H., Huo N., Gu Y.Q., Steinwand M.A. (2009). Development of SSR markers and analysis of diversity in Turkish populations of *Brachypodium distachyon*. BMC Plant Biol..

[25] Hussain B. (2015). Modernization in plant breeding approaches for improving biotic stress resistance in crop plants. Turkish J. Agric. For..

[26] Saade S., Maurer A., Shahid M., Oakey H., Schmöckel S.M., Negrão S., Pillen K., Tester M. (2016). Yield-related salinity tolerance traits identified in a nested association mapping (NAM) population of wild barley. Sci. Rep..

[27] Filiz E., Ozdemir B.S., Budak F., Vogel J.P., Tuna M., Budak H. (2009). Molecular, morphological and cytological analysis of diverse *Brachypodium distachyon* inbred lines. Genome.

[28] Lucas S.J., Šimková H., Šafář J., Jurman I., Cattonaro F., Vautrin S., Bellec A., Berges H., Dolezel J., Budak H. (2012). Functional features of a single chromosome arm in wheat (1AL) determined from its structure. Funct. Integr. Genom..

[29] Akpinar B.A., Lucas S., Budak H. (2017). A large-scale chromosome-specific SNP discovery guideline. Funct. Integr. Genom..

[30] Winfield M.O., Allen A.M., Burridge A.J., Barker G.L., Benbow H.R., Wilkinson P.A., Coghill J., Waterfall C., Davassi A., Scopes G. (2016). High-density SNP genotyping array for hexaploid wheat and its secondary and tertiary gene pool. Plant Biotechnol. J..

[31] Lucas S.J., Salantur A., Yazar S., Budak H. (2017). High-throughput SNP genotyping of modern and wild emmer wheat for yield and root morphology using a combined association and linkage analysis. Funct. Integ. Genom..

[32] Peleg Z., Cakmak I., Ozturk L., Yazici A., Jun Y., Budak H., Korol A.B., Fahima T., Saranga Y. (2009). Quantitative trait loci conferring grain mineral nutrient concentrations in durum wheat× wild emmer wheat RIL population. Theor. App. Genet..

[33] Leonforte A., Sudheesh S., Cogan N.O., Salisbury P.A., Nicolas M.E., Materne M., Forster J.W., Kaur S. (2013). SNP marker discovery, linkage map construction and identification of QTLs for enhanced salinity tolerance in field pea (*Pisum sativum* L.). BMC Plant. Biol..

[34] Lorieux M. (2012). MapDisto: Fast and efficient computation of genetic linkage maps. Mol. Breed..

[35] Masoudi B., Mardi M., Hervan E.M., Bihamta M.R., Naghavi M.R., Nakhoda B., Amini A. (2015). QTL mapping of salt tolerance traits with different effects at the seedling stage of bread wheat. Plant Mol. Biol. Report..

[36] Genc Y., Oldach K., Verbyla A.P., Lott G., Hassan M., Tester M., Wallwork H., McDonald G.K. (2010). Sodium exclusion QTL associated with improved seedling growth in bread wheat under salinity stress. Theor. App. Genet..

[37] Gupta P.K., Balyan H.S., Gahlaut V., Kulwal P.L. (2012). Phenotyping, genetic dissection and breeding for drought and heat tolerance in common wheat: Status and prospects. Plant Breed. Rev..

[38] Budak H., Hussain B., Khan Z., Ozturk N.Z., Ullah N. (2015). From genetics to functional genomics: Improvement in drought signaling and tolerance in wheat. Front. Plant Sci..

[39] Sheoran S., Malik R., Narwal S., Tyagi B.S., Mittal V., Kharub A.S., Tiwari V., Sharma I. (2016). Genetic and molecular dissection of drought tolerance in wheat and barley. J. Wheat Res..

[40] Salvi S., Tuberosa R. (2015). The crop QTLome comes of age. Curr. Opi. Biotec..

[41] Shukla S., Singh K., Patil R.V., Kadam S., Bharti S., Prasad P., Singh N.K., Khanna-Chopra R. (2015). Genomic regions associated with grain yield under drought stress in wheat (*Triticum aestivum* L.). Euphytica.

[42] Kirigwi F.M., Van Ginkel M., Brown-Guedira G., Gill B.S., Paulsen G.M., Fritz A.K. (2007). Markers associated with a QTL for grain yield in wheat under drought. Mol. Breed..

[43] Pinto R.S., Reynolds M.P., Mathews K.L., McIntyre C.L., Olivares-Villegas J.J., Chapman S.C. (2010). Heat and drought adaptive QTL in a wheat population designed to minimize confounding agronomic effects. Theor. Appl. Genet..

[44] Quarrie S.A., Pekic Quarrie S., Radosevic R., Rancic D., Kaminska A., Barnes J.D., Leverington M., Ceoloni C., Dodig D. (2006). Dissecting a wheat QTL for yield present in a range of environments: From the QTL to candidate genes. J. Exp. Bot..

[45] Golabadi M., Arzani A., Mirmohammadi Maibody S.A.M., Tabatabaei B.E.S., Mohammadi S.A. (2011). Identification of microsatellite markers linked with yield components under drought stress at terminal growth stages in durum wheat. Euphytica.

[46] Lopes M.S., Reynolds M.P., McIntyre C.L., Mathews K.L., Jalal Kamali M.R., Mossad M., Feltaous Y., Tahir I.S.A., Chatrath R., Ogbonnaya F. (2013). QTL for yield and associated traits in the Seri/Babax population grown across several environments in Mexico, in the West Asia, North Africa and South Asia regions. Theor. Appl. Genet..

[47] Maccaferri M., Sanguineti M.C., Corneti S., Ortega J.L.A., Ben Salem M., Bort J., DeAmbrogio E., Del Moral L.F.G., Demontis A., El-Ahmed A. (2008). Quantitative trait loci for grain yield and adaptation of durum wheat (*Triticum durum* Desf.) across a wide range of water availability. Genetics.

[48] Salem K.F.M., Roder M.S., Borner A. (2007). Identification and mapping quantitative trait loci for stem reserve mobilisation in wheat (*Triticum aestivum* L.). Cereal Res. Commun..

[49] Bennett D., Izanloo A., Reynolds M., Kuchel H., Langridge P., Schnurbusch T. (2012). Genetic dissection of grain yield and physical grain quality in bread wheat (*Triticum aestivum* L.) under water-limited environments. Theor. Appl. Genet..

[50] Kumar S., Sehgal S.K., Kumar U., Prasad P.V.V., Joshi A.K., Gill B.S. (2012). Genomic characterization of drought tolerance-related traits in spring wheat. Euphytica.

[51] Myles S., Peiffer J., Brown P.J., Ersoz E.S., Zhang Z., Costich D.E., Buckler E.S. (2009). Association mapping: Critical considerations shift from genotyping to experimental design. Plant Cell.

[52] Crossa J., Burgueno J., Dreisigacker S., Vargas M., Herrera-Foessel S.A., Lillemo M., Singh R.P., Trethowen R., Warburton M., Franco J. (2007). Association analysis of historical bread wheat germplasm using additive genetic covariance of relatives and population structure. Genetics.

[53] Reynolds N., Latos P., Hynes-Allen A., Loos R., Leaford D., O’Shaughnessy A., Mosaku O., Signolet J., Brennecke P., Kalkan T. (2012). NuRD suppresses pluripotency gene expression to promote transcriptional heterogeneity and lineage commitment. Cell Stem Cell.

[54] Heffner E.L., Sorrells M.E., Jannink J.L. (2009). Genomic selection for crop improvement. Crop Sci..

[55] Korte A., Farlow A. (2013). The advantages and limitations of trait analysis with GWAS: A review. Plant Meth..

[56] Shu Y., Yu D., Wang D., Bai X., Zhu Y., Guo C. (2012). Genomic selection of seed weight based on low-density SCAR markers in soybean. Genet. Mol. Res..

[57] Jannink J.L., Lorenz A.J., Iwata H. (2010). Genomic selection in plant breeding: From theory to practice. Brief. Func. Genom..

[58] Nadaf J., Riggio V., Yu T.P., Pong-Wong R. (2012). Effect of the prior distribution of SNP effects on the estimation of total breeding value. BMC Proc..

[59] Ogutu J.O., Schulz-Streeck T., Piepho H.P. (2012). Genomic selection using regularized linear regression models: Ridge regression, lasso, elastic net and their extensions. BMC Proc..

[60] Mochida K., Yamazaki Y., Ogihara Y. (2004). Discrimination of homoeologous gene expression in hexaploid wheat by SNP analysis of contigs grouped from a large number of expressed sequence tags. Mol. Genet. Genom..

[61] Leader D.J. (2005). Transcriptional analysis and functional genomics in wheat. J. Cereal Sci..

[62] Close T.J., Wanamaker S.I., Caldo R.A., Turner S.M., Ashlock D.A., Dickerson J.A., Wing R.A., Muehlbauer G.J., Kleinhofs A., Wise R.P. (2004). A new resource for cereal genomics: 22K barley GeneChip comes of age. Plant Physio..

[63] Ueda A., Kathiresan A., Bennett J., Takabe T. (2006). Comparative transcriptome analyses of barley and rice under salt stress. Theor. App. Genet..

[64] Walia H., Wilson C., Condamine P., Ismail A.M., Xu J., Cui X., Close T.J. (2007). Array-based genotyping and expression analysis of barley cv. Maythorpe and Golden Promise. BMC Genom..

[65] Kim D.Y., Hong M.J., Jang J.H., Seo Y.W. (2012). cDNA-AFLP analysis reveals differential gene expression in response to salt stress in *Brachypodium distachyon*. Genes Genom..

[66] Garg B., Puranik S., Misra S., Tripathi B.N., Prasad M. (2013). Transcript profiling identifies novel transcripts with unknown functions as primary response components to osmotic stress in wheat (*Triticum aestivum* L.). Plant Cell Tissue Organ Cult..

[67] Meng C., Quan T.Y., Li Z.Y., Cui K.L., Yan L., Liang Y., Dai J.L., Xia J.M., Liu S.W. (2017). Transcriptome profiling reveals the genetic basis of alkalinity tolerance in wheat. BMC Genom..

[68] Poersch-Bortolon L.B., Pereira J.F., Nhani Junior A., Gonzáles H.H.S., Torres G.A.M., Consoli L., Arenhart R.A., Bodanese-zanettini M.H., Margis-Pinheiro M. (2016). Gene expression analysis reveals important pathways for drought response in leaves and roots of a wheat cultivar adapted to rainfed cropping in the Cerrado biome. Genet. Mol. Biol..

[69] Ma J., Li R., Wang H., Li D., Wang X., Zhang Y., Zhen W., Duan H., Yan G., Li Y. (2017). Transcriptomics analyses reveal wheat responses to drought stress during reproductive stages under field conditions. Front. Plant Sci..

[70] Goyal E., Amit S.K., Singh R.S., Mahato A.K., Chand S., Kanika K. (2016). Transcriptome profiling of the salt-stress response in *Triticum aestivum* cv. Kharchia Local. Sci. Rep..

[71] Haque E., Kawaguchi K., Komatsu S. (2011). Analysis of proteins in aerenchymatous seminal roots of wheat grown in hypoxic soils under waterlogged conditions (supplementary material). Protein Pept. Lett..

[72] Kong F.J., Oyanagi A., Komatsu S. (2010). Cell wall proteome of wheat roots under flooding stress using gel-based and LC MS/MS-based proteomics approaches. Biochim. Biophys. Acta.

[73] Alvarez S., Roy Choudhury S., Pandey S. (2014). Comparative quantitative proteomics analysis of the ABA response of roots of drought-sensitive and drought-tolerant wheat varieties identifies proteomic signatures of drought adaptability. J. Prot. Res..

[74] Caruso G., Cavaliere C., Foglia P., Gubbiotti R., Samperi R., Laganà A. (2009). Analysis of drought responsive proteins in wheat (*Triticum durum*) by 2D-PAGE and MALDI-TOF mass spectrometry. Plant Sci..

[75] Demirevska K., Zasheva D., Dimitrov R., Simova-Stoilova L., Stamenova M., Feller U. (2009). Drought stress effects on Rubisco in wheat: Changes in the Rubisco large subunit. Acta Physiol. Plant..

[76] Kamal A.H.M., Cho K., Choi J.S., Jin Y., Park C.S., Lee J.S., Woo S.H. (2013). Patterns of protein expression in water-stressed wheat chloroplasts. Biol. Plant..

[77] Jiang S.S., Liang X.N., Li X., Wang S.L., Lv D.W., Ma C.Y., Li X.H., Ma W.J., Yan Y.M. (2012). Wheat drought-responsive grain proteome analysis by linear and nonlinear 2-DE and MALDI-TOF mass spectrometry. Int. J. Mol. Sci..

[78] Ford K.L., Cassin A., Bacic A.F. (2011). Quantitative proteomic analysis of wheat cultivars with differing drought stress tolerance. Front. Plant Sci..

[79] Zhang M., Lv D., Ge P., Bian Y., Chen G., Zhu G., Li X., Yan Y. (2014). Phosphoproteome analysis reveals new drought response and defense mechanisms of seedling leaves in bread wheat (*Triticum aestivum* L.). J. Prot..

[80] Yang F., Jørgensen A.D., Li H., Søndergaard I., Finnie C., Svensson B., Jiang D., Wollenweber B., Jacobsen S. (2011). Implications of high-temperature events and water deficits on protein profiles in wheat (*Triticum aestivum* L. cv. *Vinjett*) grain. Proteomics.

[81] Hurkman W.J., Vensel W.H., Tanaka C.K., Whitehand L., Altenbach S.B. (2009). Effect of high temperature on albumin and globulin accumulation in the endosperm proteome of the developing wheat grain. J. Cereal Sci..

[82] Kamal A.H.M., Cho K., Kim D.E., Uozumi N., Chung K.Y., Lee S.Y., Choi J.S., Cho S.W., Shen C.S., Woo S.H. (2012). Changes in physiology and protein abundance in salt-stressed wheat chloroplasts. Mol. Biol. Rep..

[83] Gao L., Yan X., Li X., Guo G., Hu Y., Ma W., Yan Y. (2011). Proteome analysis of wheat leaf under salt stress by two-dimensional difference gel electrophoresis (2D-DIGE). Phytochemistry.

[84] Jacoby R.P., Millar A.H., Taylor N.L. (2010). Wheat mitochondrial proteomes provide new links between antioxidant defense and plant salinity tolerance. J. Prot. Res..

[85] Delisle G., Champoux M., Houde M. (2001). Characterization of oxalate oxidase and cell death in Al-sensitive and tolerant wheat roots. Plant Cell Physiol..

[86] Oh M.W., Roy S.K., Kamal A.H.M., Cho K., Cho S.W., Park C.S., Choi J.S., Komatsu S., Woo S.H. (2014). Proteome analysis of roots of wheat seedlings under aluminum stress. Mol. Biol. Rep..

[87] Li G., Peng X., Xuan H., Wei L., Yang Y., Guo T., Kang G. (2013). Proteomic analysis of leaves and roots of common wheat (*Triticum aestivum* L.) under copper-stress conditions. J. Proteome Res..

[88] Kamal A.H.M., Cho K., Komatsu S., Uozumi N., Choi J.S., Woo S.H. (2012). Towards an understanding of wheat chloroplasts: A methodical investigation of thylakoid proteome. Mol. Biol. Rep..

[89] Kim K.H., Kamal A.H.M., Shin K.H., Choi J.S., Heo H.Y., Woo S.H. (2010). Large-scale proteome investigation in wild relatives (A., B and D genomes) of wheat. Acta Biochim. Biophys. Sin..

[90] Wang Y., Hu H., Xu Y., Li X.X., Zhang H.J. (2011). Differential proteomic analysis of cadmium-responsive proteins in wheat leaves. Biol. Plant..

[91] Cailin G.E., Yan D.I.N.G., Zegang W.A.N.G., Dingzhen W.A.N., Yulong W.A.N.G., Shang Q., Shishi L.U.O. (2009). Responses of wheat seedlings to cadmium, mercury and trichlorobenzene stresses. J. Environ. Sci..

[92] Bino R.J., Hall R.D., Fiehn O., Kopka J., Saito K., Draper J., Nikolau B.J., Mendes P., Roessner-Tunali U., Beale M.H. (2004). Potential of metabolomics as a functional genomics tool. Trends Plant Sci..

[93] Saito K., Matsuda F. (2010). Metabolomics for functional genomics, systems biology and biotechnology. Ann. Review Plant Biol..

[94] Saito K., Hirai M.Y., Yonekura-Sakakibara K. (2008). Decoding genes with coexpression networks and metabolomics–‘majority report by precogs’. Trends Plant Sci..

[95] Yuan J.S., Galbraith D.W., Dai S.Y., Griffin P., Stewart Jr C.N. (2008). Plant systems biology comes of age. Trends Plant Sci..

[96] Fernie A.R. (2003). Metabolome characterization in plant system analysis. Funct. Plant Biol..

[97] Yonekura-Sakakibara K., Saito K. (2006). Review: Genetically modified plants for the promotion of human health. Biotech. Lett..

[98] Hong J., Yang L., Zhang D., Shi J. (2016). Plant metabolomics: An indispensable system biology tool for plant science. Inter. J. Mol. Sci..

[99] Wen W., Li K., Alseekh S., Omranian N., Zhao L., Zhou Y., Xiao Y., Jin M., Ying N., Liu H. (2015). Genetic determinants of the network of primary metabolism and their relationships to plant performance in a maize recombinant inbred line population. Plant Cell.

[100] Kusano M., Saito K. (2012). Role of metabolomics in crop improvement. J. Plant Biochem. Biotech..

[101] Hall R., Beale M., Fiehn O., Hardy N., Sumner L., Bino R. (2002). Plant metabolomics: The missing link in functional genomics strategies. Plant Cell.

[102] Fernie A.R., Schauer N. (2009). Metabolomics-assisted breeding: A viable option for crop improvement. Trends Genet..

[103] Matsuda F., Hirai M.Y., Sasaki E., Akiyama K., Yonekura-Sakakibara K., Provart N.J., Sakurai T., Shimada Y., Saito K. (2010). AtMetExpress development: A phytochemical atlas of *Arabidopsis* development. Plant Physiol..

[104] Lei Z., Huhman D.V., Sumner L.W. (2011). Mass spectrometry strategies in metabolomics. J. Biol. Chem..

[105] Tohge T., Fernie A.R. (2009). Web-based resources for mass-spectrometry-based metabolomics: A user’s guide. Phytochemistry.

[106] Afendi F.M., Okada T., Yamazaki M., Hirai-Morita A., Nakamura Y., Nakamura K., Ikeda S., Takahashi H., Altaf-ul Amin M., Darusman M.K. (2011). KNApSAcK family databases: Integrated metabolite–plant species databases for multifaceted plant research. Plant Cell Physiol..

[107] Pandey M.K., Roorkiwal M., Singh V.K., Ramalingam A., Kudapa H., Thudi M., Chitikineni A., Rathore A., Varshney R.K. (2016). Emerging genomic tools for legume breeding: Current status and future prospects. Front. Plant Sci..

[108] Urano K., Kurihara Y., Seki M., Shinozaki K. (2010). ‘Omics’ analyses of regulatory networks in plant abiotic stress responses. Curr. Opin. Plant Biol..

[109] Obata T., Fernie A.R. (2012). The use of metabolomics to dissect plant responses to abiotic stresses. Cell. Mol. Life Sci..

[110] Zivy M., Wienkoop S., Renaut J., Pinheiro C., Goulas E., Carpentier S. (2015). The quest for tolerant varieties: The importance of integrating “omics” techniques to phenotyping. Front. Plant Sci..

[111] Endo T.R., Gill B.S. (1996). The deletion stocks of common wheat. J. Heredity.

[112] Erayman M., Sandhu D., Sidhu D., Dilbirligi M., Baenziger P.S., Gill K.S. (2004). Demarcating the gene-rich regions of the wheat genome. Nucleic Acids Res..

[113] Francki M.G., Hayton S., Gummer J., Rawlinson C., Trengove R.D. (2016). Metabolomic profiling and genomic analysis of wheat aneuploid lines to identify genes controlling biochemical pathways in mature grain. Plant Biol. J..

[114] Michaletti A., Naghavi M.R., Toorchi M., Zolla L., Rinalducci S. (2018). Metabolomics and proteomics reveal drought-stress responses of leaf tissues from spring-wheat. Sci. Rep..

[115] Salt D.E., Baxter I., Lahner B. (2008). Ionomics and the study of the plant ionome. Annu. Rev. Plant Biol..

[116] Shelden M.C., Roessner U. (2013). Advances in functional genomics for investigating salinity stress tolerance mechanisms in cereals. Front. Plant Sci..

[117] Satismruti K., Senthil N., Vellaikumar S., Ranjani R.V., Raveendran M. (2013). Plant ionomics: A platform for identifying novel gene regulating plant mineral nutrition. Am. J. Plant Sci..

[118] Tuberosa R. (2012). Phenotyping for drought tolerance of crops in the genomics era. Front. Physiol..

[119] Margulies M., Egholm M., Altman W.E., Attiya S., Bader J.S., Bemben L.A., Berca J., Braverman M.S., Chen Y.J., Chen Z. (2005). Genome sequencing in microfabricated high-density picolitre reactors. Nature.

[120] Mayer K.F., Rogers J., Pozniak C., Eversole K., Feuillet C., Gill B., Friebe B., Lukaszewski A.J., Sourdille P., Endo T.R. (2014). International Wheat Genome Sequencing Consortium. A chromosome-based draft sequence of the hexaploid bread wheat (*Triticum aestivum*) genome. Science.

[121] Shah T., Tayyaba A., Sadia L., Mehmood A.N. (2018). Genome editing tools: Advancing crop transformation and overview of tools. Plant Physiol. Biochem..

